# Protein structural insights into a rare PCSK9 gain-of-function variant (R496W) causing familial hypercholesterolemia in a Saudi family: whole exome sequencing and computational analysis

**DOI:** 10.3389/fphys.2023.1204018

**Published:** 2023-07-04

**Authors:** Noor Ahmad Shaik, Najla Al-Shehri, Mohammad Athar, Ahmed Awan, Mariam Khalili, Hadiah Bassam Al Mahadi, Gehan Hejazy, Omar I. Saadah, Sameer Eida Al-Harthi, Ramu Elango, Babajan Banaganapalli, Eman Alefishat, Zuhier Awan

**Affiliations:** ^1^ Department of Genetic Medicine, Faculty of Medicine, King Abdulaziz University, Jeddah, Saudi Arabia; ^2^ Princess Al-Jawhara Center of Excellence in Research of Hereditary Disorders, King Abdulaziz University, Jeddah, Saudi Arabia; ^3^ Department of Clinical Biochemistry, Faculty of Medicine, King Abdulaziz University, Jeddah, Saudi Arabia; ^4^ Department of Medical Genetics, Faculty of Medicine, Umm Al-Qura University, Makkah, Saudi Arabia; ^5^ Science and Technology Unit, Umm Al-Qura University, Makkah, Saudi Arabia; ^6^ Department of Pharmacology, College of Medicine, Khalifa University, Abu Dhabi, United Arab Emirates; ^7^ Department of Research and Development, Al Borg Medical Laboratories, Jeddah, Saudi Arabia; ^8^ Department of Pediatrics, Pediatric Gastroenterology Unit, Faculty of Medicine, King Abdulaziz University, Jeddah, Saudi Arabia; ^9^ Department of Clinical Pharmacology, Faculty of Medicine, King Abdulaziz University, Jeddah, Saudi Arabia; ^10^ Department of Biopharmaceutics and Clinical Pharmacy, Faculty of Pharmacy, The University of Jordan, Amman, Jordan; ^11^ Center for Biotechnology, Khalifa University, Abu Dhabi, United Arab Emirates

**Keywords:** familial hypercholesterolemia, cardiovascular diseases, whole exome sequence, sanger sequence, pcsk9

## Abstract

Familial hypercholesterolemia (FH) is a globally underdiagnosed genetic condition associated with premature cardiovascular death. The genetic etiology data on Arab FH patients is scarce. Therefore, this study aimed to identify the genetic basis of FH in a Saudi family using whole exome sequencing (WES) and multidimensional bioinformatic analysis. Our WES findings revealed a rare heterozygous gain-of-function variant (R496W) in the exon 9 of the *PCSK9* gene as a causal factor for FH in this family. This variant was absent in healthy relatives of the proband and 200 healthy normolipidemic controls from Saudi Arabia. Furthermore, this variant has not been previously reported in various regional and global population genomic variant databases. Interestingly, this variant is classified as “likely pathogenic" (PP5) based on the variant interpretation guidelines of the American College of Medical Genetics (ACMG). Computational functional characterization suggested that this variant could destabilize the native *PCSK9* protein and alter its secondary and tertiary structural features. In addition, this variant was predicted to negatively influence its ligand-binding ability with *LDLR* and Alirocumab antibody molecules. This rare *PCSK9* (R496W) variant is likely to expand our understanding of the genetic basis of FH in Saudi Arabia. This study also provides computational structural insights into the genotype-protein phenotype relationship of *PCSK9* pathogenic variants and contributes to the development of personalized medicine for FH patients in the future.

## 1 Introduction

Familial hypercholesterolemia (FH) is a globally underdiagnosed genetic condition characterized by life-long elevation of low-density lipoprotein cholesterol (LDL-C) in plasma (≥190 mg/dL) (Alhabib et al., 2021). FH results from inherited pathogenic mutations in genes that regulate hepatic LDL-C clearance and cholesterol metabolism (Mlinaric et al., 2020; Alhabib et al., 2021). FH begins to manifest at birth, and if untreated, the chronic elevation of lipids in the blood leads to plaque formation in arterial walls, subsequently accelerating atherosclerosis and increasing the risk of developing premature coronary artery disease (Alallaf et al., 2017; Hooper et al., 2018). Early detection and therapeutic intervention are critical to delay or prevent cardiovascular morbidity and mortality in patients with FH (Blanco-Vaca et al., 2018).

Majority of clinically diagnosed FH patients carry autosomal dominant loss-of-function (LOF) mutations in the low-density lipoprotein receptor (*LDLR*) and apolipoprotein-B (*APOB*) genes or gain-of-function (GOF) mutations in the proprotein convertase subtilisin/kexin type 9 (*PCSK9*) gene (Alallaf et al., 2017; Awan et al., 2019; Al-Waili et al., 2020). A rare form of autosomal recessive FH caused by loss-of-function (LOF) mutations in the low-density lipoprotein receptor adaptor protein 1 (*LDLRAP1*) gene has been reported (Paththinige et al., 2017; Chan et al., 2019). Interestingly, most of the mutations occur in *LDLR* (80%), whereas only 5% occur in APOB and 3% occur in *PCSK9* (Alallaf et al., 2017). Recent evidence supports the presence of other genes with numerous pathogenic variants, with either a causative or contributory role in FH pathogenesis. These genes include adenosine triphosphate-binding cassette transporters G5 and G8 (*ABCG5* and *ABCG8*), lipase A (*LIPA*), apolipoprotein E (*APOE*), signal transducing adaptor family member 1 (*STAP1*), cholesteryl ester transfer protein (*CETP*), and sterol regulatory element binding transcription factor 2 (*SREBP2*) (de Grooth et al., 2004; Paththinige et al., 2017; Blanco-Vaca et al., 2018; Ibrahim et al., 2021).

The current global prevalence of FH is estimated to be 1:300 (Beheshti et al., 2020), and its occurrence among founder populations and specific ethnicities is even higher (Alallaf et al., 2017; Sturm et al., 2018). This is particularly true for genetically isolated populations, such as in Saudi Arabia, where the high rate of consanguineous marriage plays a significant role in the higher incidence of genetic disorders within the population (Alallaf et al., 2017; Awan et al., 2019a). As a culturally distinct population, Saudi Arabia has been reported to have a 3-fold higher prevalence of FH (Awan et al., 2022). However, genetic data on FH, particularly founder FH mutations, among Saudi Arabian patients is scarce (Sturm et al., 2018; Al-Allaf et al., 2017; Alallaf et al., 2017; Awan et al., 2021b). In recent years, few whole exome sequencing (WES) and target gene panel testing-based studies have reported a few rare variants in LDLR (Awan et al., 2022; Chan et al., 2018; Beheshti et al., 2020) and APOB (Banaganapalli et al., 2017; Awan et al., 2021a)genes, however, the role of PCSK9 gene variants in Saudi FH patients is not yet reported.

Molecular-level understanding and functional characterization of the effect of variants on candidate proteins are essential for the development of suitable therapeutic strategies (Banaganapalli et al., 2017). However, the experimental characterization of every variant is a time-consuming, expensive, and complex process that requires a variety of skills and infrastructure. However, the expansion of computational biology applications and artificial intelligence algorithms in genomics has led to the development of various web servers and software that can perform better molecular, structural, and functional analyses of plausible disease-causing genes and variants within a short time with limited resources (Awan et al., 2021a). For example, graph-based algorithms have been shown to aid in disease-causative gene selection by removing irrelevant and redundant genes using different criteria (Harling-Lee et al., 2022) (Saberi-Movahed et al., 2022). Although numerous FH-causative LDLR, APOB, and PCSK9 variants have been reported in the literature, detailed bioinformatics-based genotype-protein phenotype characterization is lacking (Awan et al., 2019a; Meshkov et al., 2021; Guo et al., 2020a).

The main goal of this study is to identify the pathogenic variant causing FH in Saudi families through whole exome sequencing. Moreover, this study also aims to utilize computational biology methods to understand the relationship between variant macromolecular structures and their function, and role in FH.

## 2 Materials and methods

### 2.1 Recruitment of FH patients and their families

In this study, a Saudi Arabian family was clinically examined in the dyslipidemia clinic at King Abdulaziz University Hospital (KAUH) using the combined Simon Broome Register and the Dutch Lipid Clinic Network (DLCN) criteria for FH diagnosis. The family was then referred for molecular testing. This research work was approved by the Institutional Research Ethics Committee. All participants analyzed in this study gave their written and informed consent after briefing them about the study design, potential risks of any discomfort, and benefits. After careful interviews with the family members, collected a detailed family history of FH and other cardiovascular abnormalities. Plasma lipid profile data (LDL-C, high-density lipoprotein cholesterol (HDL-C), triglycerides, and total cholesterol) of the family were collected from health records. For molecular testing, approximately 5 mL (ml) of venous blood samples from whole family and 200 Saudi control participants were collected. All control subjects had a normal lipid profile (triglycerides <1.70 mmol/L, total cholesterol <5 mmol/L, HDL-C 1.40–1.55 mmol/L, and LDL-C 2.50–4.11 mmol/L) as per their health records. They were recruited after thoroughly inquiring about their clinical and family history during personal interviews.

### 2.2 Molecular genetic analysis

#### 2.2.1 DNA isolation

DNA (deoxyribonucleic acid) was isolated from white blood cells of the peripheral blood samples following the manufacturer’s protocol (Haven Scientific DNAbler-Blood Kit), and then the purity and concentration of DNA based on optical density ratios of 260/230 and 260/280 were assessed using Nanodrop (DeNovix DS-11 Series) Spectrophotometer.

#### 2.2.2 Whole exome sequencing (WES)

In brief, genomic DNA (100 ng/μl) was used to prepare DNA libraries (Ion AmpliSeq™ Library Kit 2.0), which were sequenced on the Ion GeneStudio™ S5 System with 30X coverage. All raw sequence reads were aligned against the human genome reference (GRC38, NCBI) using the Torrent Suite Software 5.4. Finally, a variant caller plug-in is used for variant calling. Then, the output data was filtered with minor allele frequency (MAF) = < 0.01 in global databases, such as the genome aggregation database (gnomAD), 1,000 genomes, and searched in local databases such as the Greater Middle Eastern Genome (GME) and the King Abdullah International Medical Research Center (KAIMRC) Genome Database for Arab population frequencies. Variants with Phred scores of >20 were selected for analysis (Alves-Bezerra and Cohen, 2017; Bener and Mohammad, 2017; Gaboon et al., 2020; Kamar et al., 2021) (Supplementary Figure S1). From this list, heterozygous variants in the coding regions of known FH causative genes were retained for downstream analyses.

#### 2.2.3 Variant validation and segregation analysis using dideoxy sequencing method

The WES identified a potential FH variant which was further validated using the deoxy—Sanger sequencing method. In this context, oligonucleotide primer sets (forward:5′-TGTTCTTTAAGCCCTCCTCTC-3′ and reverse:5′-AGAGCTGGAGTCTGGAGGAT-3′) spanning at least 100 bp upstream and downstream of the variant location were designed using the Primer-BLAST website hosted by the National Center for Biotechnology Information (NBCI) (https://www.ncbi.nlm.nih.gov/tools/primer-blast/). These primers were tested using the PCR primer stats tool (Supplementary Table S1) (https://www.bioinformatics.org/sms2/pcr_primer_stats.html) to assess the melting temperature (>50C), guanine-cytosine (GC) ratio (>40%), primer length (18–22 bp), self-annealing, and hairpin loop formation parameters. The target gene region was amplified by polymerase chain reaction (PCR) (Veriti™ 96-Well Thermal Cycler, Applied Biosystems, United States) and bidirectionally sequenced using the Sanger method (Applied Biosystems SeqStudio Genetic Analyzer) with both forward and reverse primers. To identify and annotate the variant position and nature, the generated sample sequences were aligned against the reference candidate gene sequence using the BioEdit 7.2 computational program. Based on the sequencing results, we were able to determine variant segregation patterns among the family members and control subjects (Awan et al., 2022).

### 2.3 Bioinformatic analysis of FH variant

#### 2.3.1 Sequence based annotation

Variant pathogenicity was determined based on the prediction scores of SIFT (Sorting Intolerant from Tolerant), PolyPhen2 (Polymorphism Phenotyping), and Loss-of-Function Tool (loFtool) computational tools. In this context, we entered query variant details such as reference mRNA sequence or nucleotide sequence position in the Variant Effect Predictor (VEP) tool hosted on the Ensembl web server. From the VEP output, we selected the prediction scores of the SIFT, PolyPhen-2, and loFtools. In addition, the variant was queried on the Franklin webserver (https://franklin.genoox.com/clinical-db/home) to classify it based on the ACMG criteria with the existing population, computational, functional, segregation, *de novo*, and allelic data (Harrison et al., 2019).

#### 2.3.2 Structure based annotations

##### 2.3.2.1 3D protein modeling

The crystal structure of PCSK9 (PDB code 2P4E, resolution 1.98) was initially obtained for molecular modeling from the Protein Data Bank (PDB). The Swiss model server was used to build mutant models using the PCSK9 crystal structure as a template, and the quality of the generated model was assessed using the global model quality estimation (GMQE) score. In addition, an artificial intelligence program named AlphaFold generated 3D structure was used to improve the accuracy and dependability of the protein structure predictions.

AlphaFold, a DeepMind advanced deep learning system, predicts protein folding and generates accurate 3D protein structure models by combining deep neural networks and unique computational approaches. The AlphaFold models were compared to the PCSK9 crystal structure and mutant models created with the Swiss model server. To evaluate the stereochemical quality of the wild-type and mutant models, we used multiple programs, including Procheck, Verify3D, and ERRAT (https://saves.mbi.ucla.edu/), as reported by Laskowski et al. (1996). Additionally, the altered models were improved using Swiss PDB Viewer 3.5 and energy minimization (steepest descent). Finally, using PyMOL software, all of the created protein models, including those generated by AlphaFold, were displayed and analyzed (Grell et al., 2006).

##### 2.3.2.2 Secondary structure and protein stability analysis

The secondary structures of wild-type and mutant proteins were generated using the PDBsum web server with amino acid coordinates as inputs to analyze the differences in their secondary structures, such as α-helices, β-pleated sheets, and loops. The impact of the genetic variant on the stability of the corresponding protein was analyzed using the Multi-AgEnt Stability pRedictiOn (MAESTRO) web server by providing 3D structures of both native and variant protein forms (Laimer et al., 2015; Awan et al., 2021a).

##### 2.3.2.3 Molecular docking with LDLR and monoclonal antibody

The ClusPro server, which relies on the Fast Fourier Transform (FFT) algorithm-based docking program (PIPER16) (Kozakov et al., 2006), was used to perform molecular docking between *LDLR* (PDB ID: 3P5B) and the query protein. This docking step provides ten candidate models for both proteins (wildtype and mutant), with cluster scores ranging from 0 to 9 as the best values, ranking them from the heaviest “0″cluster size to the lightest “9"(Kozakov et al., 2017; Vajda et al., 2017; Desta et al., 2020). For antibody docking, Alirocumab (CID 88214187), a fully human monoclonal antibody, was used to perform molecular docking with query PCKS9, in both native and mutant conditions, using the DockThor webserver, the blind docking approach with grid centers on the x, y, and z-axes of 37.42, 24.71, and 34.51, respectively, with a maximum grid size of 40 for the three-dimensional grid (Reyes-Soffer et al., 2017). This web server relies on phenotypic crowding-based multiple solution steady-state genetic algorithms that adhere to the following parameters: 24 docking runs, 1,000,000 evaluations per docking run, a population of 1,000 individuals, and a maximum of 100 leaders on each docking procedure. The affinity of the interaction between the query protein and ligand molecules was represented in the form of a total energy (Etotal) score that calculates the sum of the van der Waals and electrostatic potentials between the 1–4 atom pairs (Guedes et al., 2021).

## 3 Results

### 3.1 Clinical presentation

**FIGURE 1:**
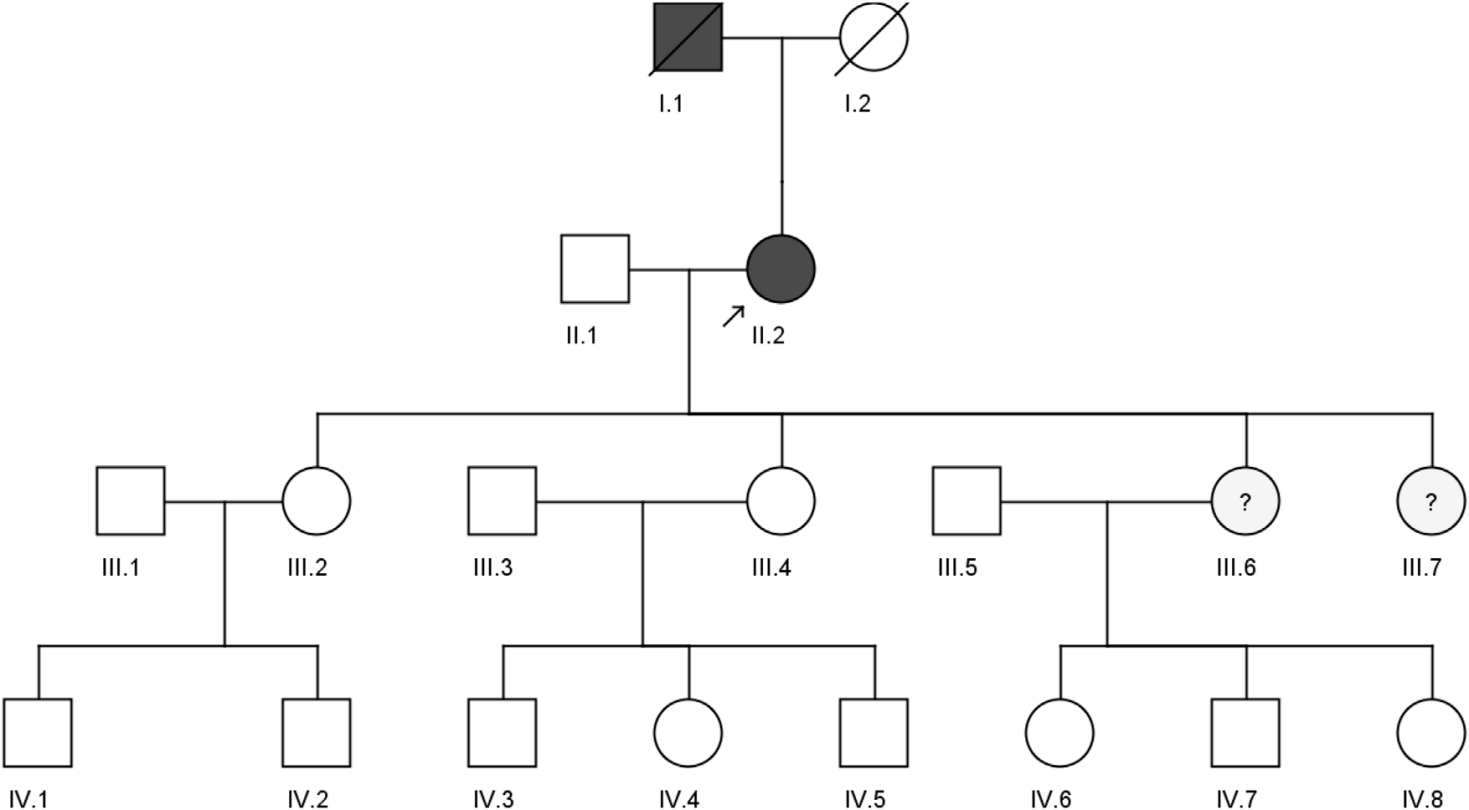
Pedigree of a four-generation Saudi family with a clinically diagnosed FH patient. The arrow indicates the index case, which was screened by both whole exome sequencing and sanger sequencing, while II.1, III.2, and III.4 were screened by the sanger sequencing method.

A 59-year-old woman from the western region of Saudi Arabia was clinically diagnosed with FH in 2018 with a positive family history (II.2). She initially visited the KAUH emergency room with a complaint of shortness of breath. She was diagnosed with coronary ischemia and referred to the dyslipidemia clinic, where she was treated for hypercholesterolemia, since her late twenties. She had smoked for 30 years, had a corneal wheel in her eyes, and no Achilles tendon growth was found (Figure 1). She was clinically diagnosed as heterozygous FH (HeFH). Her (I.1) father had premature coronary artery disease (CAD) and died in his 40s. However, we could not access any of his clinical records. The biochemical tests of the proband (II.2) show high levels of LDL-C (225 mg/dl), total cholesterol (355.2 mg/dl), and triglycerides (142.6 mg/dl), with a normal HDL-C level (66.6 mg/dl). She had been on statins (atorvastatin/20 g per day), which inhibit cholesterol synthesis, since her clinical diagnosis. No evidence of xanthomas (cholesterol deposits) was found on her tendons, elbows and knees. She is the mother of four daughters: III.2 (39 years old), III.4 (37 years old), III.6 (21 years old), and III.7 (16 years old). Despite having a high lipid profile, two sisters (III.6 and III.7) refused genetic testing due to privacy concerns. Furthermore, we could not test the offspring of III.2 (IV.1 and IV.2), III.4 (IV.3, IV.4, and IV.5), and III.6 (IV.6, IV.7, and IV.8) because of the lack of interest from those families. The abnormal plasma lipid profiles of III.6 and III.7 suggest that they are likely to have familial hypercholesterolemia (FH). They may have inherited the disease from their mother, II.2, in an autosomal dominant manner. However, this family is lost to clinical follow-up; hence, we could not ascertain our clinical assumptions and their current health statuses.

### 3.2 Genetic analysis

#### 3.2.1 Whole exome sequence (WES) results

The exome sequencing of the proband generated approximately 67,610 variants, of which only approximately 20,202 were localized in the coding region. These variants were filtered based on: (i) mapping to known FH causative genes (*LDLR, APOB, PCSK9, LDLRAP1, APOE, ABCG5, ABCG8,* and *LIPA*); (ii) zygosity; (iii) minor allele frequency of less than 1% (MAF <0.01) in the global population databases; and (iv) Phred score of >20. Based on the filtering criteria mentioned above, we identified a rare heterozygous missense variant at c.1486C>T (rs374603772) in the *PCSK9* gene in exon 9, causing a substitution of the amino acid residue R to W at the 496th position in the protein. In the gnomAD database, the global MAF of this variant was 0.00004 (12 heterozygotes of 139,471 individuals). In South Asian populations MAF is 0.0002 (8/15,254), and 0.00003 (4/63,319) in Europeans (non-Finnish). Surprisingly, this variant has not yet been reported in other global populations. Additionally, this variant was not detected in the 9497 exomes of GME or the 1,563 exomes of the KAIMRC database, the majority of them Arab nationals, if not Saudi Arabians.

#### 3.2.2 Sanger sequencing results

**FIGURE 2:**
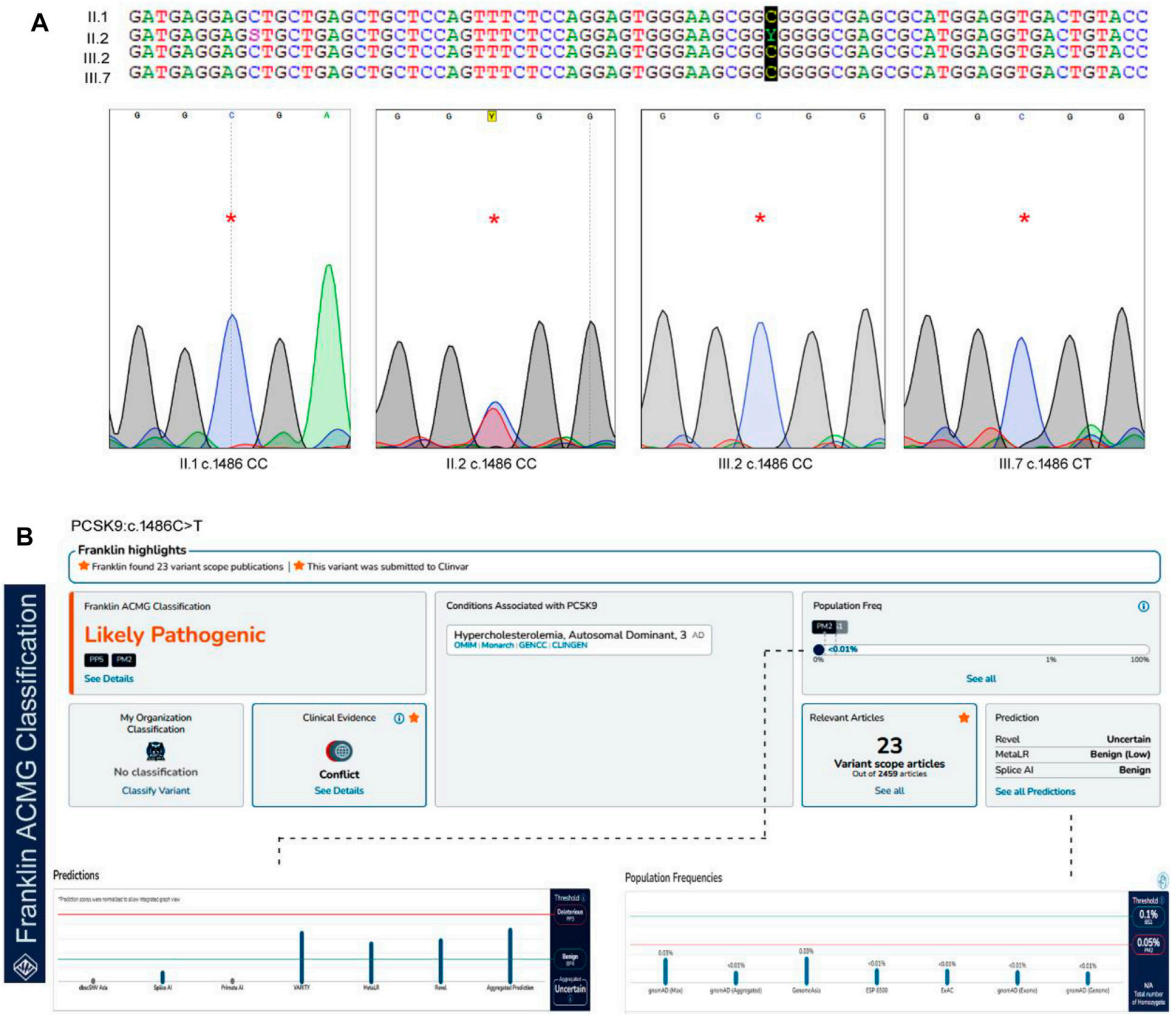
The sequence analysis of PCSK9, c.1486C>T variant. **(A)**. The genotypes and the chromatograms of the screened family members using sanger sequencing method, **(B)**. Pathogenicity prediction scores for PCSK9 variant in Franklin server by ACMG Classification (https://franklin.genoox.com/clinical-db/home) showing the likely pathogenic prediction under PP5 classification.

The Sanger sequencing results of the family confirmed that the proband (II.2) was a heterozygous carrier of the c.1486C>T variant, but none of the screened family members (II.1, III.2, and III.4), nor the 200 healthy control samples had this variant (Figure 2). We could not confirm the variant segregation pattern in this family because the rest of the family members refused to participate in this study, although two of the proband’s daughters (III.6 and III.7) had abnormal lipid profile that fulfilled the laboratory test diagnostic criteria for FH.

#### 3.2.3 Genotype-phenotype correlations

From the biochemical and genetic data mentioned in Table 1, we can observe that individuals with the c.1486C/T genotype (II.2) have significantly elevated cholesterol, triglycerides, and LDL-C levels compared to individuals with the c.1486C/C genotype (II.1, III.2, and III.4). This suggests that the c.1486C/T genotype is associated with a more severe lipid profile in the proband. However, further analysis and larger sample sizes are needed to draw more definitive conclusions about the genotype-phenotype correlation.

**TABLE 1:** Biochemical findings of FH in the index case and the family members.

Family members	Genotype	Cholesterol (mg/dL)		HDL-C (mg/dL)		Triglycerides (mg/dL)		LDL-C (mg/dL)	
II.1●	c.1486C/C	101.8	Reference: below 200 mg/dL	25.4	Reference: 60 mg/dL or above	158.7	Reference: above 150 mg/dL	45	Reference: below 100 mg/dL
II.2*●	c.1486C/T	355.2		66.6		318		225	
III.2●	c.1486C/C	178.1		47.1		75.8		116	
III. 4●	c.1486C/C	188.6		67.1		55.8		140	
III.6	-	219.2		47.17		32.5		272.4	
III.7	-	296.7		51.58		97.5		243.32	

Note: LDL-C: Low-density lipoprotein cholesterol, TC: total cholesterol, TG: triglycerides, HDL-C: High-density lipoprotein cholesterol. * Exome sequenced individuals. ● Sanger sequenced individuals.

### 3.3 Bioinformatic analysis

#### 3.3.1 Pathogenicity prediction

In our study, we utilized two widely used computational tools, SIFT and PolyPhen2, to assess the potential impact of the c.1486C>T variant on protein function. SIFT predicts the tolerance of an amino acid substitution based on sequence conservation, while PolyPhen2 predicts the potential pathogenicity of a variant by considering multiple sequence and structure-based features. The c.1486C>T variant was assigned a SIFT prediction value of 0.08, which suggests that it is a tolerated variant. On the other hand, PolyPhen2 assigned a prediction value of 0.798 to the variant, indicating a higher likelihood of being deleterious. This discrepancy in the predictions suggests the need for further evaluation and interpretation. In our whole-exome analysis, we incorporated these SIFT and PolyPhen2 predictions to prioritize and annotate variants. The predictions from these tools provided valuable information regarding the potential functional impact of the c.1486C>T variant. However, it is important to note that computational predictions alone may not provide conclusive evidence and require independent evaluation. According to the ACMG guidelines, this variant is likely to be pathogenic under the PP5 classification. PP5 criteria refer to pathogenic variants, that require independent evaluation. This variant is extremely rare in the GnomAD databases, but it has previously been reported in the ClinVar and Uniprot databases with no supporting clinical evidence (Harrison et al., 2019).

#### 3.3.2 3D protein modeling and secondary structure

Procheck Ramachandran plot analysis of the wild-type and mutant PCKS9 protein models showed that approximately 99.8% and 99.4% of the amino acids fell in the allowed regions, while 0.2% and 0.6% were in the disallowed regions, respectively. The overall structural quality of the protein models was confirmed using the ERRAT (Wild-type, 91.818; Mutant, 87.037) and Verify3D (Wildtype, 95.5% A.A. 3D score of = 0.2; Mutant, 94.2% A.A. 3D score of = 0.2) scores (Supplementary Figure S2; Supplementary Table S2). Secondary structure analysis was performed using the PDBSUM web server on Swiss modeller built *PCSK9* molecular models (wild-type and mutant forms) with a GQMEN score of >0.82. The native *PCSK9* secondary structure is characterized by 12 beta chains, 1 beta-alpha-beta unit, 6 beta hairpins, 9 beta bulges, 38 strands, 14 helices, 7 helix-helix interactions, 47 beta tuns, 7 gamma turns, and 12 disulfide bonds. In comparison to the native PCSK9, the R496W variant carrying protein has lost the 1 sheet and 1 helix-helix interaction but gained 2 beta hairpins, 3 beta bulges, 3 strands, 2 helices, 14 beta turns, and 3 gamma turns (Table 2). These findings suggest that the R496W variant of PCSK9 introduces structural alterations in the protein. The loss of a beta sheet and helix-helix interaction indicates potential disruptions in the overall protein folding and stability. On the other hand, the gained beta hairpins, beta bulges, strands, helices, beta turns, and gamma turns suggest that the mutation could influence the local structural elements and potentially affect the protein’s functional properties.

**TABLE 2:** The difference secondary structure elements SSE of the wildtype and mutant PCSK9 proteins.

Secondary structure elements SSE	Wildtype PCSK9 protein	Mutant PCSK9 protein
	Total	Total
Beta Sheets	12	10
Beta alpha beta units	1	1
Beta hairpins	6	7
Beta bulges	9	9
Strands	38	36
Helices	14	13
Helix-Helix interacts	7	5
Beta turns	47	57
Gamma turns	7	9
Disulphide bond	12	12

#### 3.3.3 Protein stability

We analyzed the changes in the stability of the mutant *PCKS9* protein using the MAESTRO webserver. This computational program relies on statistical scoring functions (SSFs) and different machine learning approaches to quantify the extent of protein destabilization based on free energy values (ΔΔGpred). ΔΔGpred values below 0.0 indicate a stable protein, whereas if above 0.0 indicate an unstable protein. The ΔΔGpred value of the R496W variant was −0.032 kcal/mol, suggesting that R-to-W substitution may destabilize the PCSK9 protein. The confidence estimation score (Cpred) of this prediction was 0.93. On a scale of 0.0–1.0, any Cpred score closer to 1 corresponds to a perfect consensus of all prediction (support vector machines, artificial neural networks, and multiple linear regression agents) agents. (Laimer et al., 2015).

#### 3.3.4 Molecular docking between *PCSK9* and *LDLR*

The ClusPro software provided the cluster energy scores for ten docking models of the two proteins complexes, incorporating *LDLR* as the receptor and each of wildtype and mutant PCSK9 as ligands. The resulting complex of wild-type *PCSK9* and *LDLR* complex has 23 hydrogen bonds between *LDLR* and the wild-type *PCSK9* protein. The number of hydrogen bonds between *LDLR* and the mutant *PCSK9* protein was reduced to 15 (Supplementary Table S3). The cluster energy value increased from −1,340.4 kJ/mol in the wildtype to −1,214.6 kJ/mol in the mutant *PCSK9*-*LDLR* complex, when focusing on the selected models (o), and the best candidate docking models (Supplementary Table S3). The molecular complexes of *LDLR* and *PCKS9* were stabilized by the formation of >20 hydrogen bonds at 3 bond distance.

The interaction between LDLR and PCSK9 is mediated by specific amino acids. In the wild-type PCSK9-LDLR complex, key amino acid interactions included Ser153(P)-Asp299, Leu298(L), Ile154(P)-Leu298(L), Pro155(P)-Leu298(L), Asp238(P)-Asn295(L), Ile369(P)-Asn301(L), Ser372(P)-Leu318(L), Try374(P)-Leu318(Cys319, Pro320(L)), Cys375(P)-Leu318(L), Thr377(P)-Asn309(Asp310, Cys308(L)), Cys378(P)-Leu318(Val307, Cys308(L)), Phe379(P)-Val307(Cys308, Asn301, His306(L)), and Val380(P)-His306(L). In the mutant PCSK9-LDLR complex, the key amino acid interactions were found to be Glu84(P)-Lys811(L), Ser89(P)-Gln242(L), Arg93(P)-Gln242(L), Arg96(P)-Ser244(L), Arg104(P)-His769(L), Gly106(P)-Leu772(L), Gln254(P)-His87(L), Val277(P)-Lys283(L), Arg476(P)-Phe801(L), Pro479(P)-Asn812(L), Glu482(P)-Lys816(L), Gln554(P)-His837(L), Gln555(P)-Glu835(L), Thr573(P)-Asp748, Thr749(L), and His602(P)-Asp834(L).

These detailed amino acid interactions provide insights into the specific residues involved in the interaction between LDLR and PCSK9. The changes in hydrogen bonding and amino acid interactions in the mutant PCSK9-LDLR complex compared to the wild-type complex may have implications for the stability and functionality of the complex.

#### 3.3.5 Molecular docking with alirocumab

The DockThor webserver was used to perform molecular docking between the antibody Alirocumab and the *PCSK9* protein (wildtype and mutant). The protein-antibody docking prediction values represented as total energy (Etotal), which is the sum of the van der waals and electrostatic potential between protein-ligand atom pairs and torsion term of the ligand (Santos et al., 2020) (Guedes et al., 2021). The docking of wildtype *PCSK9* protein and the Alirocumab complex had a total energy of −64.497 kJ/mol (Etotal) with a score of −5.934 kJ/mol, while the mutant-monoclonal antibody complex had a total energy of −62.084 kJ/mol with a score of −6.839 kJ/mol (Table 3; Supplementary Tables S4, S5).

**TABLE 3:** The docking scores calculated from the total energies of wildtype PCSK9, mutant PCSK9 docking with Alirocumab.

Name	Score	Total energy kJ/mol	Van der waals energy kJ/mol	Electrostatic energy kJ/mol
Wildtype PCSK9-Alirocumab	−5.934	−64.49	0.84	−40.48
Mutant PCSK9-Alirocumab	−6.839	−62.08	−5.27	−31.99

## 4 Discussion

In this study, we detected a rare heterozygous c.1486C>T p. (R496W) gain of function variant in the exon 9 of the PCSK9 gene in the proband (II.2), which was not found in the screened healthy family members or the 200 healthy normolipidemic Saudi Arabian controls. The *PCSK9* variants are usually classified into 2 types: gain-of-function (GOF) and loss-of-function (LOF), where the GOF variant causes a decrease in *LDLR* on hepatocytes, leading to the phenotypes of FH, whereas the LOF is involved in the low LDL-C levels that lower the risk of developing coronary heart disease with no known adverse effects on human health (Bayona et al., 2020; Guo et al., 2020b).

The c.1486C>T variant was predicted to be a likely pathogenetic (PP5) mutation according to the ACMG guidelines. These guidelines propose a five-tier categorization to evaluate the Mendelian disease variants into “pathogenic" (P), “likely pathogenic" (LP), “uncertain significance" (VUS), “likely benign" (LB), and “benign" (B). This classification considers allele frequency data, reputable sources, functional and computational data, case-level data, and nature and location of the variants (Harrison et al., 2019). The c.1486C>T variant was reported in FH patients from Italy (Pisciotta et al., 2006), Japan (Ohta et al., 2016), Spain (Ibarretxe et al., 2018), and Turkey (Eroğlu, 2018). All these studies have performed detailed clinical characterization of the c.1486C>T but did not report its functional impact on protein structure and function.

Furthermore, our findings suggest that c.1486C>T is an FH causative variant, as it was not found in Saudi and Middle Eastern regional population genetic databases like GME and KAIMRC and has a very low frequency of 12 het carriers in 139,471 exomes of the gnomAD (Table 4). Eight of these carriers were from South Asia (of 15,254 exomes) and 4 among the non-Finnish Europeans (of 63,319 exomes).

**TABLE 4:** A list of FH causative PCSK9 variants reported from Middle Eastern countries.

No.	Country	Variant ID	Chromosomal location	Coding regions change	Amino acid change	FH zygosity	Reference
1	Saudi Arabia	-	1:55,512,313	c.517delC	p.P173fs	HeFH	Al-Allaf et al. (2015)
2		rs509504	1:55,523,033	c.1026A>G	p.Q342H	HoFH	
3		rs540796	1:55,524,197	c.1380A>G	p.Y243C	HoFH	
4		rs562556	1:55,524,237	c.1420G>A	p.V474I	HoFH	
5		-	1:55,529,113	c.1935delG	p.L645fs	HeFH	
6		rs505151	1:55,529,187	c.2009G>A	p.G670E	HoFH	
7	Tunisia	rs505151	1:55,529,187	c.2009G>A	p.G670E	HoFH	Alhababi and Zayed (2018)
8		rs533273863	1: 55046643	c.520C>T	p.P174S	HeFH	
9		-	-	c.1545T > G	p.F515L	HeFH	Slimani et al. (2015)
10		rs72555377	1:55,039,880	c.65_66insGCTGCT	p.L22_L23dup	HeFH	
11	Lebanon	rs72555377	1:55,039,880	c.65_66insGCTGCT	p.L22_L23dup	HeFH	Abifadel et al. (2009)
12		rs11591147	1: 55,039,447	c.137G>T	pR46L	HeFH	Bamimore et al. (2015)
13	Oman	rs562556	1: 55058564	c.1420G>A	I474V	HeFH	Al-Waili et al. (2013)

PCSK9 is a proteolytic enzyme and a member of the proprotein convertase family of serine proteases, that is mainly expressed in the liver (Guo et al., 2020b). The GOF variant c.1486C>T (p.R496W) is located in the C-terminal’s first domain, which is the second reported domain with the highest GOF variants, as the majority of which are mapped to the subtilisin-like catalytic region of the Peptidase S8 domain, whereas the majority of LOF variants are in both regions of the Peptidase S8 domain (Bayona et al., 2020). Furthermore, variant c.1486C>T is a missense mutation that results in the substitution of arginine (R), an amino acid with a positively charged side chain, with tryptophan (W), a hydrophobic, aromatic side chain, in codon 496, resulting in different secondary structure elements (SSE), such as the absence of two beta sheets, two strands, one helix, and two helix-helix interactions, while gaining one beta-hairpin and two gamma turns. As a result, this mutation alters the folding pattern, exhibiting tremendous conformational changes in the tertiary structure by the absence of the protein’s two-chain structure (chain P-chain A) into a malfunctioning protein with highly unstable *PCSK9* (Shaik and Banaganapalli, 2019; Awan et al., 2021a).

The role of the *PCSK9* GOF variants in accumulating the cholesterol in the plasma was first reported in 2003 (Abifadel et al., 2003). It is responsible for the degradation of *LDLR* on the cell membrane of the hepatocytes by binding to the extracellular domain of LDLR (Awan et al., 2021b). Therefore, LDLR is a transmembrane receptor that binds to circulating LDL-C to form the LDLR-LDL-C complex. This complex is then internalized through clathrin-coated pits into endosomes, where the LDLR releases the LDL-C for degradation in the lysosomes and returns to the cell membrane. The prevention of LDLR recycling and hepatic LDL-C clearance by hepatocytes, and chronic elevation of LDL-C in the plasma leads to plaque formation in arterial walls, accelerates atherosclerosis, and eventually leads to the development of premature CAD (McGowan et al., 2019; Seidah and Prat, 2021).

**FIGURE 3:**
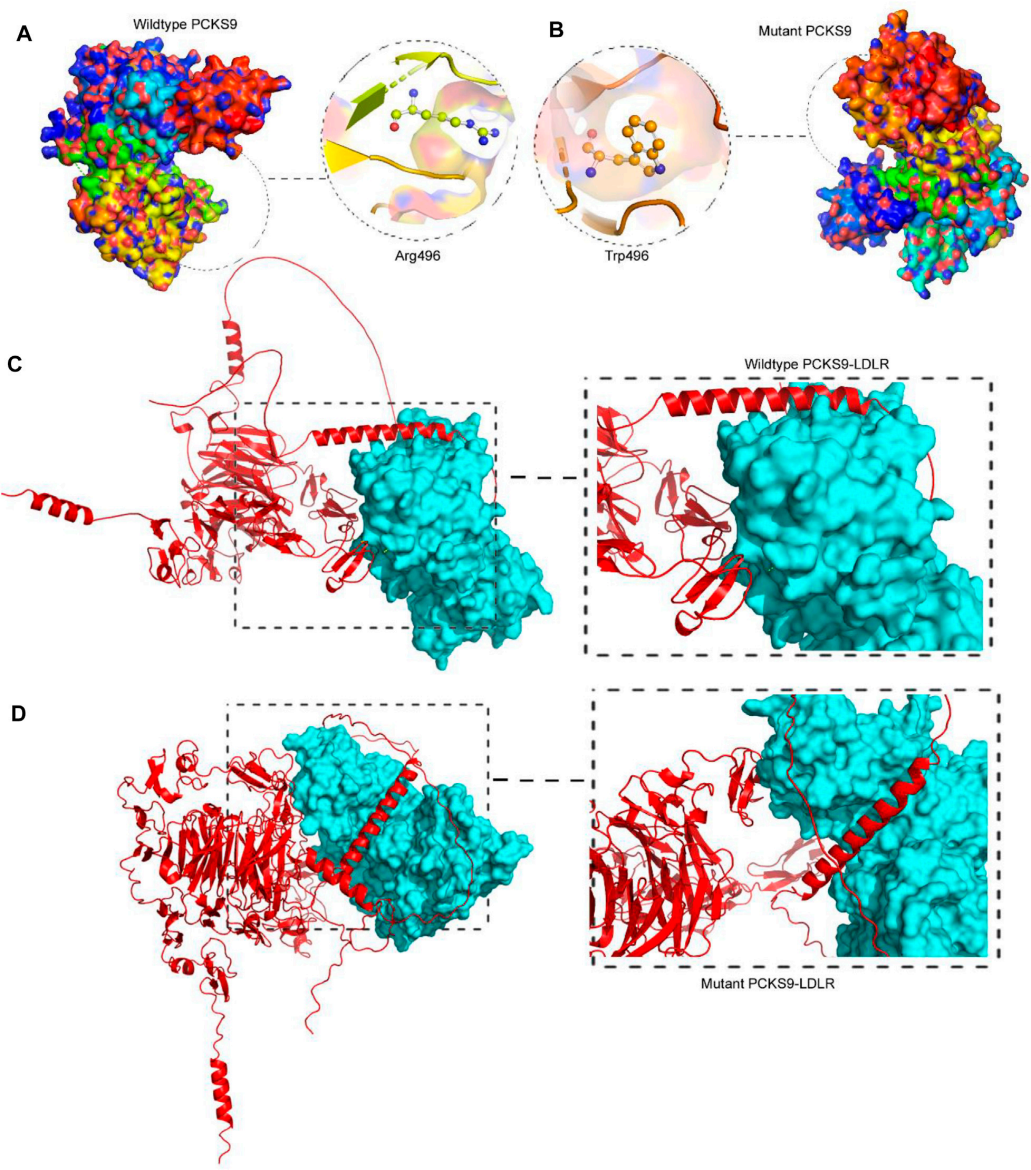
Analysis of PCSK9 tertiary structure models. **(A)**. The 3D protein model of the wildtype PCSK9 where R496 built by Swiss-Model, **(B)**. The 3D protein model of the mutant PCSK9 (496W), showing the drastic conformational changes, **(C)**. Molecular docking of wildtype PCSK9 with LDLR, **(D)**. Molecular docking of mutant PCSK9 with LDLR.

**FIGURE 4:**
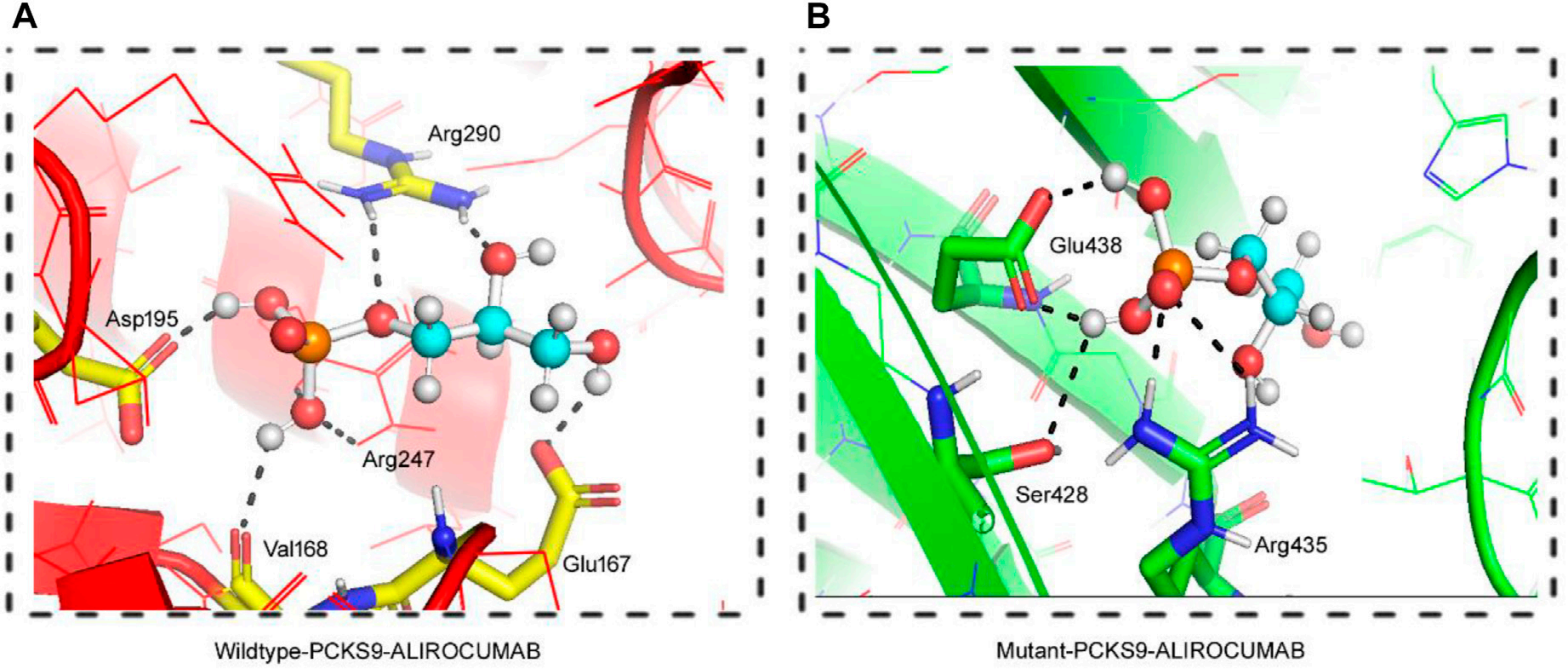
3D Visualization of the interaction between PCSK9 proteins and Alirocumab. Alirocumab with the wildtype PCSK9 protein **(A)**, and with the mutant PCSK9 protein **(B)** provided by the DockThor-VS web server, where the ball-and-stick indicating interactions of the Alirocumab with different amino acid residues in the wildtype and mutant PCSK9 proteins.

Molecular docking is the analysis of binding between biological and chemical structures using computational tools that focus on electrostatic potentials, physicochemical complementarity, and binding energy and presents them as 3D models with the best-predicted score. To compare the output of the binding of the mutant *PCSK9* with *LDLR*, molecular docking was performed between the wild-type *PCSK9* and *LDLR* as a reference. The lower energy was produced by the docking process between *PCSK9* and *LDLR,* yielding ten candidate models (Figure 3). The cluster energy score increased from −1,340.4 kJ/mol in the wildtype to −1,214.6 kJ/mol in the mutant PCSK9-LDLR complex, suggesting that the wildtype complex docked more effectively than the mutant because low binding energies correlate with higher binding affinity (Shaik and Banaganapalli, 2019; Desta et al., 2020) (Figure 4).

As previously stated, PCSK9 mutations that result in loss play an important role in lowering LDL levels in the blood. As a result, human monoclonal anti-PCSK9 antibodies have proven effective in lowering LDL-C levels and atherosclerotic cardiovascular disease (ASCVD) risk, particularly in individuals with severe phenotypes or resistance to lipid-lowering therapies (LLT). This was accomplished by binding to PCSK9 in plasma and preventing it from binding with LDLR. We performed molecular docking between both the wild type and the mutant PCSK9 and Alirocumab, a known PCSK9 inhibitor, to evaluate the change in the binding and the effectiveness of the PCSK9 inhibitor against the mutant protein. This scoring system is based on the sum of the van der Waals and electrostatic potentials of PCSK9 binding to Alirocumab (Dhankhar et al., 2020; Dhankhar et al., 2021; Dalal et al., 2022; Kumari et al., 2022). The increase in docking scores between mutant PCSK9 and Alirocumab showed a damaging effect of variant c.1486C>T on the binding potential, which decreases the Van der Waals energy, indicating molecules moving apart. This study found that the c.1486C>T variant of PCSK9 reduced the efficacy of alirocumab.

This study has few limitations. We could not recruit more families with FH to assess the role of *PCSK9* (R496W) as a hotspot mutation among Saudi Arabs. However, given the rare occurrence of PCSK9 variant-carrier FH families in any ethnic population this seems to be an unrealistic limitation. Moreover, we could not ascertain the biological impact of the *PCSK9* (R496W) variant using *in-vitro* functional biology assays because of the refusal of the participants to provide fresh tissue samples. However, to complement this lacuna, we used computational analysis as a primary functional characterization tool before undertaking technically complicated, time-consuming and expensive *in vitro* and *in vivo* methods.

In conclusion, this is the first c.1486C>T GOF variant identified in FH patients from Saudi Arabia. This study emphasizes the significance of genetic testing in identifying rare or novel FH mutations in underrepresented populations, which has the potential to reduce the burden of cardiovascular disease (CVD) in risk-group countries. Furthermore, this research provides comprehensive computational and structural insights into the genotype-protein phenotype correlation of the PCSK9 pathogenic variant with a PCSK9 inhibitor monoclonal antibody.

## Data Availability

The original contributions presented in the study are included in the article/Supplementary Material; further inquiries can be directed to the corresponding authors.
