# Supplementary material for: Protein structural insights into a rare PCSK9 gain-of-function variant (R496W) causing familial hypercholesterolemia in a Saudi family: whole exome sequencing and computational analysis
Source: Front Physiol. 2023 Jul 4;14:1204018. doi: 10.3389/fphys.2023.1204018 (PMC10353052; doi:10.3389/fphys.2023.1204018)
Supplement: Supplementary file 1 [file Table5.pdf]

H-Bonds

| Index | Residue | AA | Distance H-A | Distance D-A | Donor Angle | Protein donor? | Side chain | Donor Atom | Acceptor Atom |
|---|---|---|---|---|---|---|---|---|---|
| 1 | 435A | ARG | 2.81 | 3.67 | 146.61 | | | 5 [03] | 4053 [O2] |
| 2 | 438A | GLU | 2.11 | 2.71 | 117.78 | | | 8 [03] | 4096 [O-] |
| 3 | 438A | GLU | 3.46 | 4.01 | 115.84 | | | 4088 [Nam] | 7 [03] |
| 4 | 438A | GLU | 1.53 | 2.46 | 155.80 | | | 7 [03] | 4095 [03] |
| 5 | 504A | SER | 3.15 | 3.78 | 121.63 | | | 4676 [Nam] | 8 [03] |
| 6 | 581A | THR | 3.22 | 3.88 | 126.82 | | | 6 [03] | 5377 [03] |

Salt Bridges

| Index | Residue | AA | Distance | Protein positive? | Ligand Group | Ligand Atoms |
|---|---|---|---|---|---|---|
| 1 | 435A | ARG | 3.85 | | Phosphate | 10, 10, 4, 7, 8, 9 |

Supplementary Table S5: Types of interaction in between Mutant PCKS9 and Ligand
