# Supplementary material for: Protein structural insights into a rare PCSK9 gain-of-function variant (R496W) causing familial hypercholesterolemia in a Saudi family: whole exome sequencing and computational analysis
Source: Front Physiol. 2023 Jul 4;14:1204018. doi: 10.3389/fphys.2023.1204018 (PMC10353052; doi:10.3389/fphys.2023.1204018)
Supplement: Supplementary file 2 [file Table4.pdf]

H-Bonds

Index	Residue	AA	Distance H-A	Distance D-A	Donor Angle	Protein donor?	Side chain	Donor Atom	Acceptor Atom
1	167A	GLU	2.17	3.05	150.08	X	✓	6 [03]	1553 [O-]
2	195A	ASP	2.98	3.82	144.38	X	✓	7 [03]	1797 [0.co2]
3	195A	ASP	1.46	2.36	149.54	X	✓	8 [03]	1796 [0.co2]
4	290A	ARG	3.02	3.70	126.36	✓	✓	2655 [Ng+]	5 [03]
5	290A	ARG	1.83	2.79	158.40	✓	✓	2656 [Ng+]	5 [03]
6	308A	ARG	3.39	3.87	112.34	X	X	5 [03]	2816 [02]

Salt Bridges

Index	Residue	AA	Distance	Protein positive?	Ligand Group	Ligand Atoms
1	247A	ARG	3.93	✓	Phosphate	10, 10, 4, 7, 8, 9
2	290A	ARG	5.05	✓	Phosphate	10, 10, 4, 7, 8, 9

Supplementary Table S4: Types of interaction in between wild type PCKS9 and Ligand
