# Supplementary material for: Protein structural insights into a rare PCSK9 gain-of-function variant (R496W) causing familial hypercholesterolemia in a Saudi family: whole exome sequencing and computational analysis
Source: Front Physiol. 2023 Jul 4;14:1204018. doi: 10.3389/fphys.2023.1204018 (PMC10353052; doi:10.3389/fphys.2023.1204018)
Supplement: Supplementary file 3 [file Table1.pdf]

Supplementary Table S1: PCKS9 Primer Properties

**Statistical parameters**	**Forward primer**	**Reverse primer**
Sequence (5' -3')	TGTTCTTTAAGCCCTCCTCTC	AGAGCTGGAGTCTGGAGGAT
**Sequence length**	21	20
**GC content (%)**	47.62	55.00
**Molecular weight (Daltons)**	6274.12	6262.14
**Basic temperature Tm (^{0}C)**	52	54
**Salt adjusted temperature Tm (^{0}C)**	47	49
