# Supplementary material for: Protein structural insights into a rare PCSK9 gain-of-function variant (R496W) causing familial hypercholesterolemia in a Saudi family: whole exome sequencing and computational analysis
Source: Front Physiol. 2023 Jul 4;14:1204018. doi: 10.3389/fphys.2023.1204018 (PMC10353052; doi:10.3389/fphys.2023.1204018)
Supplement: Supplementary file 4 [file Table2.pdf]

Supplementary Table S2: PCSK9 wild and mutant protein quality scores of Procheck, Errat and Vertify-3D

Protein validation tools		PCSK9 (wildtype)	PCSK9 (mutant)
PROCHECK	Amino acids fall in the allowed region	99.8%	99.4%
	Amino acids fall in the disallowed region	0.2%	0.6%
ERRAT		91.818%	87.037%
VERTIFY-3D	Structure quality	95.5 %	94.2%
	3D score	>=0.2	>=0.2
