# Supplementary material for: Protein structural insights into a rare PCSK9 gain-of-function variant (R496W) causing familial hypercholesterolemia in a Saudi family: whole exome sequencing and computational analysis
Source: Front Physiol. 2023 Jul 4;14:1204018. doi: 10.3389/fphys.2023.1204018 (PMC10353052; doi:10.3389/fphys.2023.1204018)
Supplement: Supplementary file 5 [file Image2.pdf]

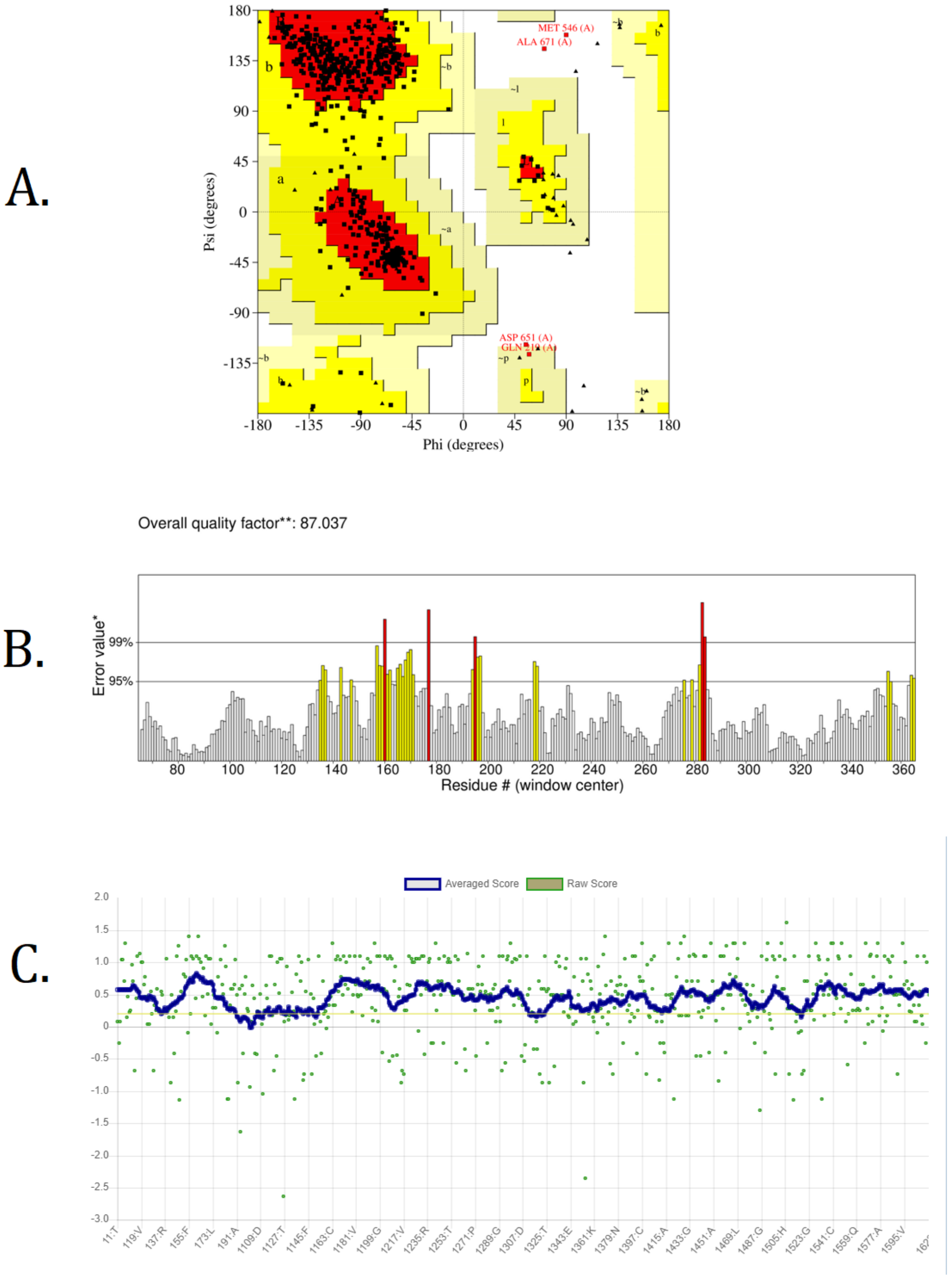

Supplementary Figure S2: .Mutant PCSK9 protein validation; A: Ramachandran plot, B: ERRAT values and C. VERIFY3D.
