# Supplementary material for: Protein structural insights into a rare PCSK9 gain-of-function variant (R496W) causing familial hypercholesterolemia in a Saudi family: whole exome sequencing and computational analysis
Source: Front Physiol. 2023 Jul 4;14:1204018. doi: 10.3389/fphys.2023.1204018 (PMC10353052; doi:10.3389/fphys.2023.1204018)
Supplement: Supplementary file 6 [file Table3.pdf]

Supplementary Table S3: wildtype and mutant LDLR-PCKS9 docking interactions by clusPro

| Protein-Ligand | Weighted Score (kJ/mol) | | Hydrogen bonds | Amino Acids interactions |
|---|---|---|---|---|
| | center | Lowest Energy | | |
| LDLR-PCSK9 (Wildtype) | -1340.4 | -1340.4 | 23 hydrogen bonds | Ser153(P)-Asp299, Leu298(L), Ile154(P)-Leu298(L), Pro155(P)- Leu298(L), Asp238(P)-Asn295(L), Ile369(P)- Asn301(L), Ser372(P)-Leu318(L), Try374(P)- Leu318, Cys319, Pro320(L), Cys375(P)- Leu318(L), Thr377(P)-Asn309, Asp310, Cys308(L), Cys378(P)-Leu318, Val307, Cys308(L), Phe379(P)- Val307, Cys308, Asn301, His306(L), Val380(P)- His306(L). |
| LDLR-PCSK9 (Mutant) | -1214.6 | -1374.3 | 15 hydrogen bonds | Glu84(P)- Lys811(L), Ser89(P)- Gln242(L), Arg93(P)- Gln242(L), Arg96(P)- Ser244(L), Arg104(P)- His769(L), Gly106(P)- Leu772(L), Gln254(P)- His87(L), Val277(P)- Lys283(L), Arg476(P)- Phe801(L), Pro479(P)- Asn812(L), Glu482(P)- Lys816(L), Gln554(P)- His837(L), Gln555(P)- Glu835(L), Thr573(P)- Asp748, Thr749(L), His602(P)- Asp834(L) |
